# MicroRNA-34 directly targets pair-rule genes and cytoskeleton component in the honey bee

**DOI:** 10.1038/srep40884

**Published:** 2017-01-18

**Authors:** Flávia C. P. Freitas, Camilla V. Pires, Charles Claudianos, Alexandre S. Cristino, Zilá L. P. Simões

**Affiliations:** 1 Faculdade de Medicina de Ribeirão Preto, Universidade de São Paulo, Ribeirão Preto, Brazil; 2 Monash Institute for Cognitive and Clinical Neuroscience, Monash University, Melbourne, Australia; 3 The University of Queensland Diamantina Institute, Translational Research Institute, Brisbane, Australia; 4 Faculdade de Filosofia, Ciências e Letras de Ribeirão Preto, Universidade de São Paulo, Ribeirão Preto, Brazil

## Abstract

MicroRNAs (miRNAs) are key regulators of developmental processes, such as cell fate determination and differentiation. Previous studies showed Dicer knockdown in honeybee embryos disrupt the processing of functional mature miRNAs and impairs embryo patterning. Here we investigated the expression profiles of miRNAs in honeybee embryogenesis and the role of the highly conserved *miR*-*34*-*5p* in the regulation of genes involved in insect segmentation. A total of 221 miRNAs were expressed in honey bee embryogenesis among which 97 mature miRNA sequences have not been observed before. Interestingly, we observed a switch in dominance between the 5-prime and 3-prime arm of some miRNAs in different embryonic stages; however, most miRNAs present one dominant arm across all stages of embryogenesis. Our genome-wide analysis of putative miRNA-target networks and functional pathways indicates *miR*-*34*-*5p* is one of the most conserved and connected miRNAs associated with the regulation of genes involved in embryonic patterning and development. In addition, we experimentally validated that *miR*-*34*-*5p* directly interacts to regulatory elements in the 3′-untranslated regions of pair-rule (*even*-*skipped, hairy, fushi*-*tarazu transcription factor 1*) and cytoskeleton (*actin5C*) genes. Our study suggests that *miR*-*34*-*5p* may regulate the expression of pair-rule and cytoskeleton genes during early development and control insect segmentation.

MicroRNAs (miRNAs) are small non-coding RNAs, 19–24 nucleotide (nt) in length, that regulate gene expression in eukaryotes by directly binding target messenger RNAs (mRNAs). Primary miRNAs are transcribed by RNA polymerase II^1^ and cleaved by the RNase III endonuclease Drosha yielding the hairpin precursor miRNA^2^. The hairpin is exported to the cytoplasm^3^ where it is cleaved by the RNase III endonuclease Dicer giving rise to mature miRNA duplex (approximately 22 nt)^4^,^5^. In most cases, one of the arms of the duplex miRNA (5p or 3p) shows higher expression levels in comparison to the oppose arm. This dominant miRNA is typically known as mature miRNA. Nonetheless, the extensive use of next generation sequencing revealed that the dominant arm can differ in different tissues and developmental times^6^,^7^,^8^,^9^,^10^ in a process called arm switching^11^. The dominant miRNA is incorporated to RNA-induced silencing complex (RISC) and direct regulation of target-genes through the action of Argonaute (AGO) protein^12^.

MiRNAs control their targets by recognizing miRNA regulatory elements (MREs) in the 3′UTR of mRNA transcripts. In most cases, the complementarity between the seed region of the miRNA (2–8 nt in the 5′end) and the mRNA is sufficient to trigger the silencing of the target transcripts^13^,^14^,^15^. In spite of that, several MREs lacking complementarity in the seed region are also known to be functional^16^,^17^,^18^. Typically miRNAs are known to be critical regulators of cell differentiation^19^ and embryonic development^20^,^21^. In addition, miRNAs have been also associated with learning and memory^22^,^23^ and several diseases, e.g. cancers^24^ and Alzheimer^25^,^26^.

In honey bee embryos, the knockdown of Dicer depletes the production of mature miRNAs leading to defects in tissue patterning and segments formation^21^. Embryonic segments are repeated body units whose formation starts just after the cellularization of blastoderm and concludes during gastrulation by an intricate regulatory network including gap, pair-rule and segment polarity genes^27^. In insects, the pair-rule genes *hairy (h*), *even*-*skipped (eve*), *runt (run*)^28^, *fushi*-*tarazu (ftz*), *ftz transcription factor 1 (ftz*-*f1*) and *wingless (wg*) are involved in the segmentation process^29^,^30^. The regulatory role of miRNAs in the segmentation process in the developing embryo is still poorly understood.

In this study, we identified 221 miRNAs in four different stages of honey bee embryogenesis, among which 97 are novel mature miRNAs. The dominance between 5p and 3p arms of a few miRNAs shifted during embryogenesis and thus support the existence of an additional mechanism involved in the arm selection other than the thermodynamic properties of miRNA duplexes. The highly conserved *miR*-*34*-*5p* is expressed throughout the embryonic development and regulates genes involved in segmentation and morphogenesis. Our results indicate a possible role of *miR*-*34*-*5p* in the regulation of early embryogenesis by directly targeting the pair-rule genes *eve, h, ftz*-*f1*, and the structural gene *actin5C (act5C*). Overall, we hypothesize that *miR*-*34*-*5p* regulates the expression of the pair rule genes and *act5C* in the developing embryo and thus, participates in the determination of cell fate in segmentation process and in the migration of germ-layer forming-cells during embryonic development in insects.

## Results

### Transcriptional profile revealed novel miRNAs and arm switching in honey bee embryogenesis

**Figure 1:**
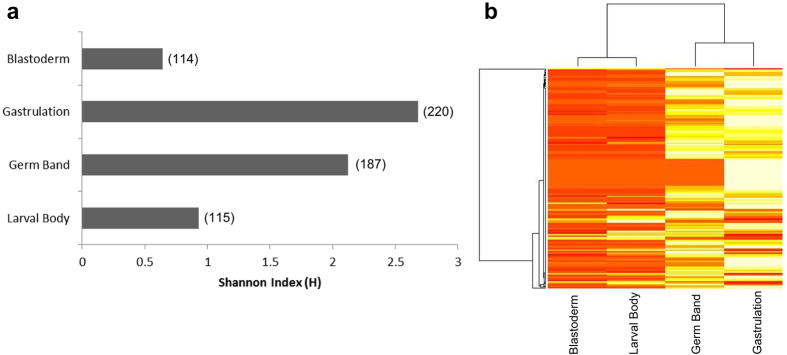
Diversity of miRNAs expressed in blastoderm, gastrulation, germ band and larval body in honey bee embryogenesis. (**a**) Diversity of Shannon index for each developmental stage showed that the highest diversity of miRNAs was found in gastrulation stage. (**b**) Hierarchical clustering of the expressed miRNAs in honey bee embryogenesis revealed similar expression profiles between germ band and gastrulation and between blastoderm and larval body.

We performed high-throughput sequencing of small RNAs extracted from embryos at different developmental stages: blastoderm (18–24 hours), gastrulation (32–38 hours), germ band (42–48 hours), and larval body (66–72 hours). The sequence length distribution of mapped reads revealed that 95 to 97% corresponds to 19 to 24 nt long reads, consistent with the canonical length of mature miRNAs (Supplementary Fig. S1). In total, 221 mature miRNAs (originated from 127 different precursor miRNAs) are expressed in honey bee female embryos. The insect-specific *ame*-*miR*-*306*-*5p* and the conserved *ame*-*miR*-*184*-*3p*, and *ame*-*miR*-*92c*-*3p* are highly expressed in all embryonic stages (Supplementary Table S1). The highest diversity of miRNA regulation was found in gastrulation and germ band stages (Shannon entropy 2.68 and 2.12, respectively) (Fig. 1a). The hierarchical clustering analysis showed similar results, as gastrulation was grouped with germ band and blastoderm with larval body (Fig. 1b).

**Figure 2:**
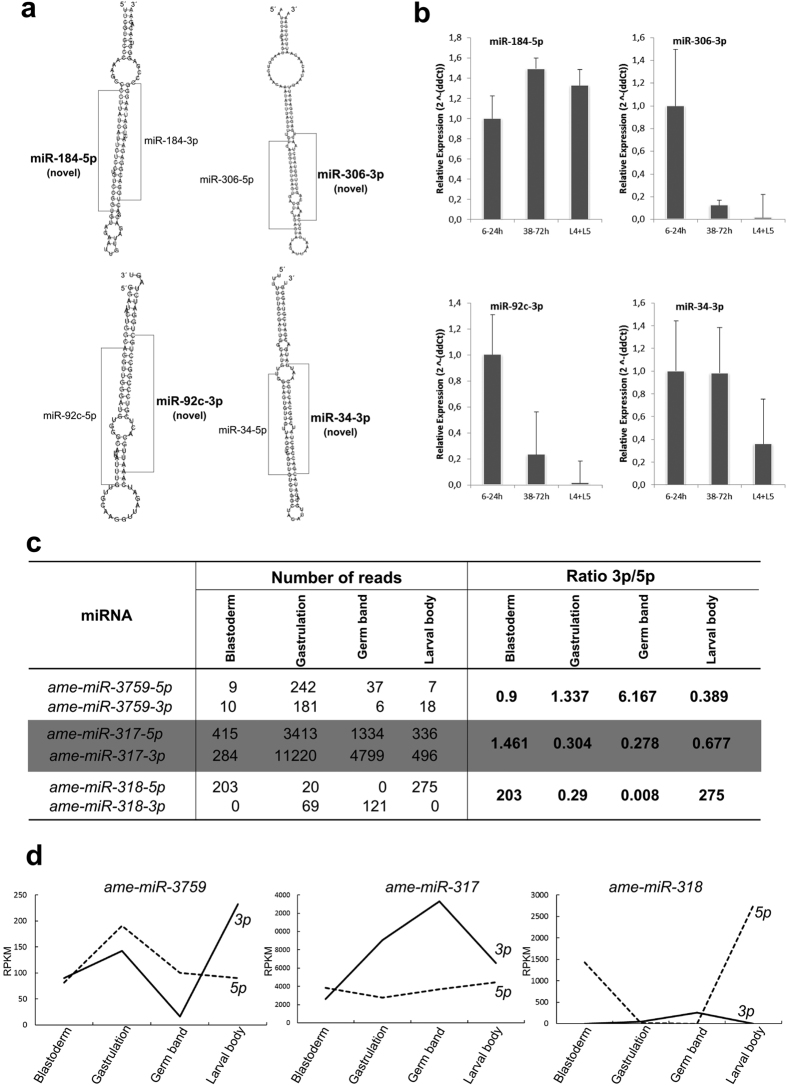
Identification of novel miRNAs and evidence for miRNA arm switch in *A. mellifera* embryogenesis. (**a**) Localization of novel miRNAs in the precursor sequences. Secondary structure of honey bee miRNAs precursors (retrieved from miRBase version 20) were generated by RNAfold. Mature miRNAs 3p and 5p are highlighted in the secondary structure. (**b**) Amplification of novel miRNAs by RT-qPCR. cDNA from female embryo and worker larvae were used to validate the novel miRNAs found in the small RNAs libraries of honey bee embryos. (**c**) Table of number of reads mapped to each arm of miRNAs that showed arm switching during honey bee embryogenesis (**d**) Expression profiles of *ame*-*miR*-*3759, ame*-*miR*-*317* and *ame*-*miR*-*318* showed alternated dominance of the 5p and 3p arms in honey bee embryogenesis.

Among the 221 mature miRNAs, 97 correspond to arms from previously known miRNA precursors that have not been found expressed in honey bees; for this reason, they are hereafter called novel mature (Supplementary Table S2). The position occupied by the novel miRNAs in the precursor sequence is consistent to functional mature molecules, as they localize in the stem region of the hairpin structure (Fig. 2a). We confirmed the transcription of four novel mature miRNA sequences (*ame*-*miR*-*184*-*5p, ame*-*miR*-*306*-*3p, ame*-*miR*-*92c*-*3p*, and *ame*-*miR*-*34*-*3p*) by qPCR from cDNA libraries of embryos (6–24 h and 38–72 h) and larvae (L4 and L5 instars) (Fig. 2b).

For 94 precursor miRNAs (74%) out of 127 expressed in honey bee embryogenesis we identified both 5p and 3p mature miRNAs were expressed at least in one stage. Most precursor miRNAs (82) had one dominant arm highly transcribed during all embryogenesis, which coincided with the mature miRNA deposited in miRBase, such as *ame*-*miR*-*34*-*5p*. Interestingly, the dominance between 5p and 3p arms of *ame*-*miR*-*3759, ame*-*miR*-*317* and *ame*-*miR*-*318* shifted during development (Fig. 2c and d). Taken together, our results suggest that both 5p and 3p arms can generate functional mature miRNAs in honey bee embryogenesis.

### *miR*-*34*-*5p* emerges as a potential regulator of developmental processes

We searched for putative miRNA-target interactions conserved between honey bees (*A. mellifera*) and fruit flies (*D. melanogaster*) and found 43460 miRNA-target interactions between 48 conserved miRNAs and 8736 conserved target genes. Among the miRNA-target interactions conserved in both species, each miRNA putatively targets 905 genes (SD ± 586 genes) in average; the highest number of targets predicted for a miRNA were 2881 genes for *miR*-*34*-*5p* and the lowest number was 44 for *miR*-*79*-*3p* (Supplementary Table S3).

**Figure 3:**
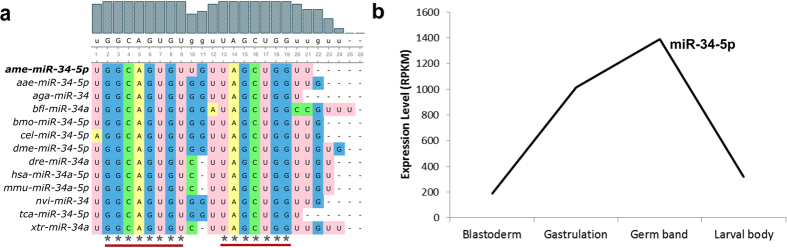
miR-34 emerges as a putative regulator of honey bee embryogenesis. (**a**) Multiple alignment of *miR*-*34*-*5p* in animal species. Gray bars indicate conservation degree for each base in the *miR*-*34*-*5p*. The nucleotide sequence underneath the bars represents the consensus sequence (the upper case letters correspond to the bases conserved in all analyzed species. The red bars indicate the conserved seed region (2–9 nt). (**b**) Transcripts levels of *miR*-*34*-*5p* in honey bee embryogenesis assessed by RNA-seq. *aee: Aedes aegypti; aga: Anopheles gambiae; ame: A. mellifera; bfl: Branchiostoma floridae; bmo: Bombyx mori; cel: Caenorhabditis elegans; dme: D. melanogaster; dre: Danio rerio; hsa: Homo sapiens; mmu: Mus musculus; nvi: Nasonia vitripennis; tca: Tribolium castaneum; xtr: Xenopus tropicalis*.

The high number of *miR*-*34*-*5p* targets conserved between *A. mellifera* and *D. melanogaster* can be explained by the high degree of conservation of *miR*-*34*-*5p* seed region (position 2–8 nt) and its complementary site (position 13–19 nt) revealed by a multiple sequence alignment (Fig. 3a).

To explore the function of the targets predicted for *miR*-*34*-*5p*, an enrichment analysis of the Gene Ontology (GO) terms was performed and revealed that *miR*-*34*-*5p* putatively regulates embryogenesis processes such as “pattern specification process” and “anatomical structure morphogenesis” (p-value = 1.57E-84 and p-value = 6.01E-43, respectively) (Supplementary Table S4). The GO term “pattern specification process” includes genes involved in segmentation process, such as the pair-rule genes *eve, ftz*-*f1, h*, and segment polarity gene *wg*. These genes are also included in the GO term “anatomical structure morphogenesis” together with *act5C*, which is a structural gene important for the cellularization of the blastoderm, an event that precedes the movements for gastrulation^31^,^32^,^33^.

The *miR*-*34*-*5p* expression profile throughout embryonic development is consistent with its putative regulatory role in the segmentation process in insect embryogenesis. Pair-rule and segment polarity genes are expressed during the transition of blastoderm to gastrulation and as a result of their expression the segments are formed by the end of gastrulation stage^31^,^32^,^33^. In honey bee embryogenesis, *miR*-*34*-*5p* reaches its highest level of expression in germ band stage (Fig. 3b; Supplementary Table S1).

Taken together, our results pointed to a regulatory role of *miR*-*34*-*5p* in the segmentation process of honey bee embryos through the regulation of the pair-rule genes *eve, ftz*-*f1, h*, the segment polarity gene *wg*, and the structural gene *act5C*.

### *miRNA*-*34*-*5p* directly targets developmental genes in honey bee embryogenesis

**Figure 4:**
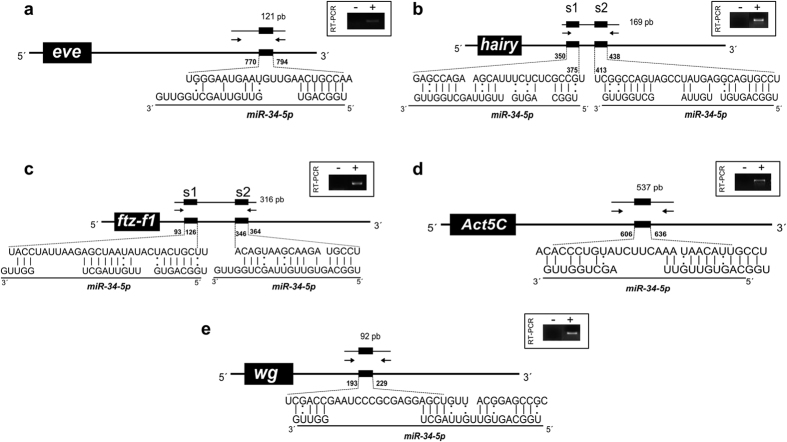
Predicted binding-sites in the developmental genes 3′UTRs for *miR*-*34*-*5p* by RNAhybrid. Schematic representation of honey bee *miR*-*34*-*5p* binding-sites and RT-PCR amplified 3′UTRs of validated genes in the dual-luciferase assay. The box in the upper-right region of each scheme contains the amplified regions and the negative control of the RT-PCR reactions. The nucleotide positions of *miR*-*34*-*5p* binding sites (small black box), numbered from the stop codon, are shown on 3′UTR feature. The arrows indicate 3′UTR regions that were PCR amplified. Perfect matches are indicated by a line, and G:U pairs by a colon.

To test if the MREs predicted in the 3′UTR of *eve, ftz*-*f1, h, wg*, and *act5C* for *miR*-*34*-*5p* are functional, we used a luciferase reporter assay. The putative *miR*-*34*-*5p* MREs identified in the 3′UTR fragment of *eve, h, ftz*-*f1, wg* and *act5C* genes were amplified from cDNA of honey bee embryos and experimentally tested using a dual-luciferase reporter (Fig. 4a–e). Only one *miR*-*34*-*5p* MRE has been predicted in the 3′UTR of *eve* gene with a perfect match to the canonical seed region (Fig. 4a). Two *miR*-*34*-*5p* MREs were predicted in *h* (Fig. 4b) and *ftz*-*f1* (Fig. 4c) 3′UTRs fragments; these MREs present mismatches in the seed region and complementarity at the 3′-compensatory site (Fig. 4b and c). The MRE predicted in the 3′UTR of *act5C* presents an extensive complementarity with *miR*-*34*-*5p* (1–13 nt, including G:U pairing) (Fig. 4d). The MRE for *miR*-*34*-*5p* in *wg* 3′UTR lacks complementarity with both seed and compensatory regions (Fig. 4e).

**Figure 5:**
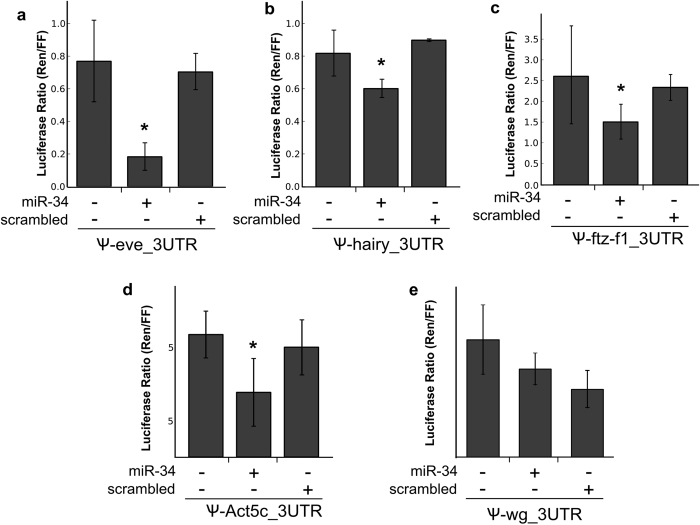
*miR*-*34*-*5p* binds to MREs in the 3′UTRs of pair-rule genes and *act5C* in honey bees. Dual-luciferase reporter analysis of *miR*-*34*-*5p* target interactions. Changes in expression ratio of Firefly (Fluc) and chimeric *Renilla* luciferase (Rluc) containing putative 3′UTR and miRNA predicted binding sites of target genes that are treated with and without *miR*-*34*-*5p* mimic and scrambled RNA are shown. Two sample *t*-tests were used to test the effect of mimic *miR*-*34*-*5p* in the honey bee 3′UTRs, *p < 0.05. The 3′UTR of *eve* (**a**) eve_3UTR; *p* = *0*.*0021*), *h* (**b**) hairy_3UTR; *p* = *0*), *ftz*-*f1* (**c**) ftz-f1_3UTR; *p* = *0*.*0097*), *act5C* (**d**) act5C_3UTR; *p* = 0.0004) are significantly down-regulated by *miR*-*34*-*5p*. The 3′UTR of *wg* (**e**) wg_3UTR; *p* = 0.0833) was down-regulated by miR-34 and also by scrambled RNA.

In the dual-luciferase assay, the fragment containing the putative MRE was inserted into a chimeric vector so that it acted as a 3′UTR of a reporter-gene (luciferase). The activity of the luciferase was measured in the presence of the mimic *miR*-*34*-*5p*, in the presence of a scrambled sequence (negative control) and in the absence of any mimic miRNA sequence. If the mimic *miR*-*34*-*5p* recognizes the putative MRE, it is expected a decrease of luciferase activity due to the prevention of the translation of luciferase transcripts. The positive-control vector (containing perfectly matched target site for *miR*-*34*-*5p*) showed significantly reduced luciferase activity in the presence of the *miR*-*34*-*5p* mimic (Supplementary Fig. S2). The tests with MREs predicted in the 3′UTR of *eve, h, ftz*-*f1*, and *act5C* also showed significant decrease (p-value < 0.05) in luciferase activity in the presence of mimic *miR*-*34*-*5p* (56, 50, 26 and 58%, respectively) (Fig. 5a–d). A reduction of 30% in luciferase activity was also observed in the *wg* assay; nonetheless, the results suggested that the fragment of *wg* 3′UTR tested in the luciferase reporter assay was also bound by the scrambled sequence (Fig. 5e). Thus, the validation assay revealed that the regulatory elements predicted for *miR*-*34*-*5p* in the 3′UTR of *eve, ftz*-*f1, h* and *act5C* are functional.

The transcript levels of *eve, ftz*-*f1, h* and *act5C* in the embryonic development were assessed by qPCR. *eve, ftz*-*f1, h* and *act5C* are expressed throughout all embryonic development and show higher levels of transcripts during blastoderm, when the cellularization process takes place and when the segmentation process begins (Supplementary Fig. S3).

## Discussion

In this study, we used high-throughput sequencing of small RNAs to identify miRNAs expressed throughout honey bee embryonic development. We found that most precursor miRNAs expressed in embryos produce mature sequences from both arms (5p and 3p) of the hairpin, with some cases of arm switching mechanism also noted. *miR*-*34*-*5p* has been identified as one of the most interesting candidates associated with developmental processes and directly targets the pair-rule genes *eve, h* and *ftz*-*f1*, and the structural gene *act5C*. This is the first time that *miR*-*34*-*5p* has been shown to bind MREs predicted in the 3′UTRs of the insect pair-rule genes and *act5C*. Our findings support the hypothesis of miRNA regulation during critical events of insect embryogenesis.

Our transcriptomics data showed that 74% of precursor miRNAs detected in different stages of the honey bee embryogenesis expressed both 5p and 3p arms. Novel mature miRNAs sequences such as *ame*-*miR*-*92c*-*3p, ame*-*miR*-*184*-*5p, ame*-*miR*-*306*-*3p*, and *ame*-*miR*-*34*-*3p* were expressed in early and late-embryogenesis as well as larval stages (Fig. 2b). In most cases, one arm showed higher expression in comparison to its opposed arm and often the dominant arm was consistent to the mature miRNA described in miRBase. The selection of a dominant arm has been attributed to the thermodynamic properties of the miRNA-5p/miRNA-3p duplex^34^,^35^. Nonetheless, an increasing number of studies has shown that the quantity of miRNAs generated from each arm can switch in different tissues and developmental stages suggesting an additional mechanism involved in arm selection^6^ possibly involving different arrangements of Argonaut and accessory proteins within the RISC complex^36^. However, the precise nature of arm switching mechanism remains to be characterized. The arm selection affects tremendously the cellular environment as each arm results in a different mature sequence targeting different set of genes^37^,^38^,^39^,^40^,^41^. Thus, different miRNA arms may be required to regulate specific target networks controlling key functional pathways in cell proliferation and differentiation during the embryogenesis.

The most highly expressed miRNAs in honey bee female embryos (i.e. *miR*-*306*-*5p, miR*-*184*-*3p* and *miR*-*92c*-*3p*) are also highly expressed in male embryos and are maternally deposited in honey bee oocytes^42^. Nonetheless, the function of these miRNAs in the insect embryogenesis remains uncertain. Interestingly, the insect-specific *miR*-*306*-*5p* is also up-regulated in ovaries of egg laying workers and queens^43^. The conserved *miR*-*184*-*3p* is also highly expressed in embryos of *Manduca sexta*^44^ and has multiple functions in female germline development and in early embryogenesis of *D. melanogaster*^45^.

The higher diversity of miRNA expression in the mid-embryogenesis, known as gastrulation and germ band stages, reflects the complexity of the morphogenetic processes that take place in these stages. The three germ layers (ecto-, meso-, and endoderm) are determined and placed in gastrulation and the cells are continuously differentiating into tissues and organs. In addition, the segments, whose domains are established by morphogenetic gradients during blastoderm^46^, can be seen in gastrulation and divide the developing insect embryo into gnathal and thoracic units^33^. Blastoderm and larval body were grouped together because the miRNAs shared by both stages are expressed in low levels (Supplementary Table S1). The lower expression levels of miRNAs observed in blastoderm is expected due to maternal-to-zygotic transition, when the maternal products are degraded, and the zygotic genome is progressively activated by the end of this stage^47^. In the larval body, the lower diversity and expression of miRNAs might be related to the fact that the boundaries of tissues and organs are already established, and the role of the miRNAs is reduced to the maintenance of tissues and organs identity.

*miR*-*34*-*5p* emerged as one of the most conserved miRNA with the highest number of predicted targets (2881 genes) corresponding to 33% of all the genes conserved between *A. mellifera* and *D. melanogaster* (8376 in total). *miR*-*34*-*5p* is highly conserved across the animal kingdom from worm to human (Fig. 3b). *miR*-*34*-*5p* plays critical role in several biological processes such as cell proliferation^48^, cell differentiation^49^, and cell cycle progression^50^. *miR*-*34*-*5p* also regulates nervous system development in vertebrates and in the fruit fly^51^,^52^. Our functional annotation analysis of the *miR*-*34*-*5p* target network indicates this miRNA as a key regulator of developmental processes such as pattern specification and morphogenesis. Among the putative targets of *miR*-*34*-*5p* are the pair-rule genes *eve, ftz*-*f1, h*, the segment polarity gene *wg*, and the structural gene *act5C*. Thus, we investigated the role of *miR*-*34*-*5p* in the segmentation genes by testing the interaction between the *miR*-*34*-*5p* and the predicted MREs in the 3′UTR of the genes *eve, ftz*-*f1, h, wg*, and *act5C* in cell culture using a dual-luciferase reporter assay.

The luciferase reporter assay showed that mimic *miR*-*34*-*5p* directly regulates *eve, ftz*-*f1, h*, and *act5C*. A detailed look at the pairing between *miR*-*34*-*5p* and its predicted MREs revealed that 3′UTRs containing both canonical seed pairing MREs (*eve* and *act5C*) and non-canonical seed pairing MREs (*ftz*-*f1* and *h*) were bound by *miR*-*34*-*5p* and seemed sufficient to repress luciferase activity. The recognition of MREs with and without canonical seed pairing by RISC is feasible due to the variable conformations that AGO can adopt to accommodate different structures of miRNA-target duplexes^36^. These findings encourage the inclusion of MREs with non-conserved seed region in the predicted target networks.

Here, we showed that the segmentation genes *eve, h* and *ftz*-*f1*, and the structural gene *act5C* are directly regulated by *miR*-*34*-*5p* in honey bee. The fact the *ftz*-*f1* is regulated by *miR*-*34*-*5p* along with genes already known to form stripes in honey bee embryos (*eve* and *h*)^28^ may indicate that this gene acts in honey bee as a pair-rule gene as it does in the beetle *Tribolium*^53^. *eve, h* and *ftz*-*f1* are primary pair-rule genes whose expression domains form periodic stripes along the anterior-posterior axis in the developing embryo positioned by overlapping gradients of maternal and gap genes in early embryogenesis^28^,^46^,^53^,^54^,^55^,^56^,^57^,^58^. Additionally, it has been postulated that miRNAs contribute to the sharpening of gene expression domains in segmentation of *D. melanogaster* embryos by creating an interface between cells expressing high levels of the target mRNA and miRNA-expressing cells with low levels of target mRNA^59^,^60^. Thus, *miR*-*34*-*5p* might contribute to the regulation of the pair-rule genes *eve, h, ftz*-*f1* during the formation of expression domains that precedes the segmentation process. Similarly, *miR*-*34*-*5p* may regulate *act5C* levels during the establishment and placement of germ layers. Together with Myosin, Actin is a major determinant of cell shape in the developing embryo. Changes in the cell shape are crucial in gastrulation when columnar cells adopt a wedge shape through apical constriction and migrate inward within the embryo and establish the mesoderm layer^61^,^62^.

Our findings point to *miR*-*34*-*5p* as novel regulatory component in the complex molecular cascade that governs insect segmentation and to a broader role of miRNAs in the early development due to the detection of mature transcripts from both 5p and 3p arms for several precursor miRNAs. Thus, this work encourages further investigation to pinpoint miRNAs as fine tuners of insect early development.

## Methods

### Sample collection and small RNAs libraries preparation

Honey bee embryos were collected from colonies kept in the apiary of the Department of Genetics at University of São Paulo (Ribeirão Preto, Brazil). To obtain controlled-aged embryos, a mated egg-laying queen was caged in a wax comb frame for six hours. Female embryos from blastoderm (18–24 h), gastrulation (32–38 h), germ band (42–48 h), and larval body (66–72 h) stages were sampled and the stages were determined according to DuPraw^33^. Total RNA was extracted from pools of 100–150 female embryos using TRIzol (Life Technologies). Concentration and purity of RNA samples were assessed by NanoDrop spectrophotometer (Thermo Scientific). Four μg of total RNA of each stage were used as template in the preparation of small RNAs libraries, performed accordingly to Illumina single-end protocol (Genome Analyzer II, Life Sciences). The libraries preparation and sequencing were performed at the High-Throughput Sequencing Facility in the University of North Carolina (Chapel Hill, USA). The libraries generated are deposited in www.ncbi.nlm.nih.gov/sra with access numbers: SRR1539264 (18–24 h), SRR3708802 (32–38 h), SRR3708803 (42–48 h), and SRR3708804 (66–72 h).

### Computational analyses of small RNA libraries

We used Cutadapt software to filter low quality reads and to remove adapter sequences from the reads for each library. Only 19–24 nt long reads were considered for further analysis. In order to identify the miRNAs expressed in honey bee embryogenesis, we used BWA^63^ to map the reads to the precursor sequences of honey bee miRNAs retrieved from miRBase (version 20). Using Python programming language (https://www.python.org/) we identified peaks of reads along the precursor sequences. The peak found in the 5′ arm corresponds to miRNA-5p; the peak found in the 3′ arm corresponds to miRNA-3p. This approach (mapping reads in miRNA precursor sequences) led to the identification of novel mature miRNAs from known miRNA precursor sequences. The calculation of Shannon entropy was performed using homemade scripts in Python.

### *In silico* identification of novel miRNAs and experimental validation by RT-qPCR

To validate our method of novel miRNAs identification, we performed both *in silico* and experimental assays for a set of selected miRNAs (*ame*-*miR*-*92c*-*3p, ame*-*miR184*-*5p, ame*-*miR*-*306*-*3p*, and *ame*-*miR*-*34*-*3p*). In the *in silico* approach, we used RNAfold^64^ to generate secondary structure of miRNAs precursors. We then identified the position occupied by the novel mature miRNA in the secondary structure to check if it was located in the stem region. The experimental approach consisted in amplifying novel miRNAs by RT-qPCR (qPCR) using cDNA of embryos in early (oocytes and embryos 0–6 h and 18–24 h old), and late (embryos 32–38 h, 42–48 h and 66–72 h old) stages of embryogenesis, and of worker larvae (stages L4 and L5). qPCR reactions were performed using both biological and technical triplicates, in 20 μl reactions: 10 μl Syber Green Master Mix 10x (Invitrogen), 1 μl cDNA, 1 μl miRNA-specific forward primer, 1 μl universal reverse primer (NCode First-strand synthesis kit, Invitrogen). The sequence of the miRNA-specific primers is listed in Supplementary Table S5. qPCR reactions were performed in the thermocycler 7500 Real Time PCR System (Applied Biosystems) with the following conditions: 2 min at 50 °C, 10 min at 95 °C followed by 40 cycles of 15 seg at 95 °C and 1 min at 60 °C. Quantification of novel miRNAs transcripts was performed according to 2^−ΔΔCt^ method^65^ and the expression of the small nuclear RNA *U5* was used as endogen control.

### Multiple sequence alignment of *miR*-*34*-*5p* sequences across metazoan

Mature sequences of *miR*-*34*-*5p* from several metazoan species were retrieved from miRBase and submitted to multiple sequence alignment using ClustalW^66^.

### Prediction of miRNA regulatory elements in honey bees and *Drosophila* 3′ UTRs

We searched for regulatory elements for miRNAs in the 3′UTRs of all predicted genes in honey bee genome using RNAhybrid^67^. To reduce the false positive miRNA-target interactions, we also conducted predictive analysis in *Drosophila* genome and considered only those interactions predicted for both species. The predicted MREs were filtered by free energy ( < −20 kcal/mol) and by p-value ( < 0.05) as a result of these filtering, MRE containing G:U pairing and interactions with non-canonical seed pairing were also considered in validation assays. Sequences for conserved miRNAs in both *Drosophila* and *A. mellifera* genomes were retrieved from miRBase (version 20) and used in target prediction analysis. The search for miRNA-regulatory elements was performed in 3′UTR (1000 nt long region downstream to the stop codon of the honey bee coding genes) of the conserved coding genes in both species.

### *In vitro* validation of *ame*-*miR*-*34*-*5p* binding sites by Dual-luciferase-reporter assays

Dual-luciferase reporter assay was performed according to Cristino, *et al*.^22^. Briefly, a 3′UTR fragment containing the predicted MRE (or a control sequence with perfect match to *miR*-*34*) was anchored to the 3′end of *Renilla reniformis* luciferase coding gene in the PsiCHECK2 vector (Promega). Cos-7 cells were co-transfected with both vector and synthetic mimic *ame*-*miR*-*34*-*5p* (miRIDIAN, Dharmacon). After 24 hours, the luciferase activity was measured using Dual Luciferase Assay (Promega), according to manufacturer’s instructions. Firefly luciferase activity was used to normalize *Renilla* luciferase activity. All transfections were performed in six replicates each time. We used two samples *t*-test to determine the difference between two conditions (*t*-test, p < 0.05). The complete method procedure used in the Luciferase-reporter assays is described in Supplementary File S1; the primers used to amplify the 3′UTR fragments and the mimic miRNAs are listed at Supplementary Table S6.

### Expression profile of *ame*-*miR*-*34*-*5p* targets in honey bee embryogenesis by qPCR

The transcript levels of *eve, h, ftz*-*f1*were assessed by qPCR in female embryos collected in blastoderm, gastrulation, germ band and larval body stages. Two micrograms of total RNA were incubated with DNase I Amplification Grade (Invitrogen) to eliminate contaminating genomic DNA (40 min at 37 °C) and inactivate the enzyme (15 min at 70 °C). First-strand cDNA was synthesized by reverse transcription using SuperScript II (Invitrogen) and oligo(dT)_12–18_ (Invitrogen). The qPCR amplification was performed using 10 μL Syber Green Master Mix (Invitrogen), 1 μl cDNA, 1 μl of both forward and reverse gene-specific primer (10 mM), 7 μl water (Supplementary Table S7). Each developmental stage was represented by three biological replicates and each replicate was tested three times (technical replicates). The qPCR was performed in a 7500 Real Time PCR System (Applied Biosystems) with the following conditions: 50 °C for 2 min and 95 °C for 10 min followed by 40 cycles of 95 °C for 15 s, and 60 °C for 1 min. Quantification of *eve, h*, and *ftz*-*f1* transcripts was performed according to 2^−ΔΔCt^ method^65^ and the expression of the ribosomal gene *rp49* was used as endogen control^68^.

## Additional Information

**How to cite this article**: Freitas, F. C. P. *et al*. MicroRNA-34 directly targets pair-rule genes and cytoskeleton component in the honey bee. *Sci. Rep.* **7**, 40884; doi: 10.1038/srep40884 (2017).

**Publisher's note:** Springer Nature remains neutral with regard to jurisdictional claims in published maps and institutional affiliations.

## Supplementary Material

Supplementary Information
