# Supplementary material for: MicroRNA-34 directly targets pair-rule genes and cytoskeleton component in the honey bee
Source: Sci Rep. 2017 Jan 18;7:40884. doi: 10.1038/srep40884 (PMC5241629; doi:10.1038/srep40884)
Supplement: Supplementary Information [file srep40884-s1.pdf]

Supplementary Information

Title: MicroRNA-34 directly targets pair-rule genes and cytoskeleton component in the honey bee.

Authors: Flávia CP Freitas, Camilla V Pires, Charles Claudianos, Alexandre dos Santos Cristino* & Zilá LP Simões*

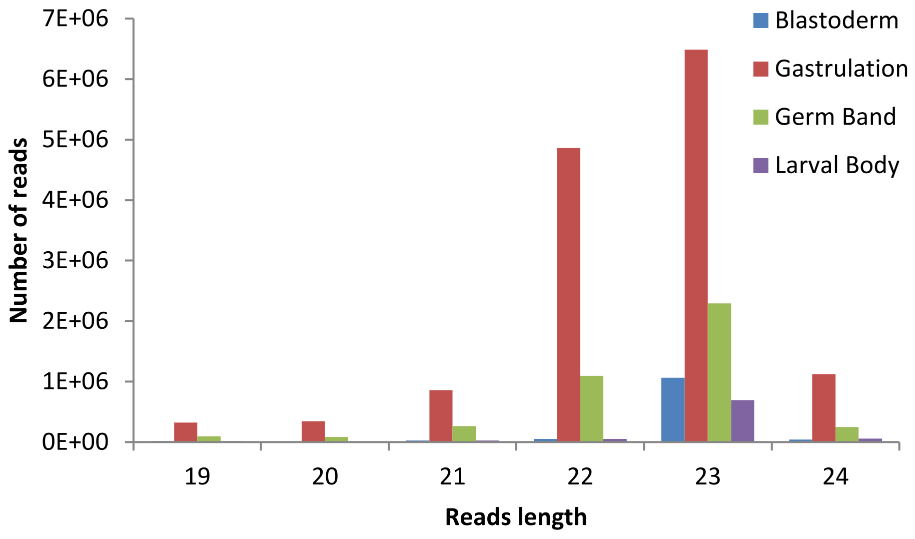

Figure S1 – Length distribution of 19-24 long reads mapped to the miRNAs precursor sequences. Most of the reads mapped to the miRNAs precursors are 22-23 nt long.

Table S1 - Expression levels of miRNAs in female honey bee embryos. The expression levels are presented as RPKM

miRNA	Blastoderm	Gastrulation	Germ band	Larval body
ame-mir-306-5p	6973073.689	2637561.374	4032002.286	6419773.37
ame-mir-92c-3p	386413.7078	404030.2712	601248.9756	630602.918

ame-mir-279b-3p	174207.9913	335004.1797	749809.6364	362985.613
ame-mir-6040-5p	162813.9115	9932.863872	35169.2804	141190.389
ame-mir-184-3p	81392.92034	1763681.933	1111865.592	117303.223
ame-mir-6051-3p	68224.47187	136616.5148	195144.0776	93316.962
ame-mir-3791-3p	62557.52126	26081.46786	72171.07012	112326.617
ame-mir-279a-3p	60301.61589	530088.0062	322989.6172	98206.3771
ame-mir-279c-3p	32859.18868	138630.1373	153090.7452	61183.0545
ame-mir-996-3p	30621.93924	156998.7459	110105.9233	43462.5206
ame-mir-92a-3p	28479.67941	78241.73463	136000.2193	43908.4974
ame-mir-263a-5p	23194.29624	852666.0565	276276.776	30662.5408
ame-mir-3747b-5p	15067.78797	34595.95752	45188.8964	30988.8672
ame-mir-100-5p	10458.03021	5798.130141	10123.56633	10460.6236
ame-mir-279c-5p	8036.059199	17585.18299	12759.23316	5718.42513
ame-mir-2944-3p	6103.973222	11749.83199	31571.96904	13801.849
ame-mir-9a-5p	5719.615088	79167.52611	52847.40307	5868.55085
ame-let-7-5p	5050.706104	10446.6498	19277.64292	8881.43756
ame-mir-2944-5p	4577.979917	883.1098705	1329.870751	3999.02162
ame-mir-2-2-5p	4541.646743	14405.55523	4830.240199	2742.23148
ame-mir-317-5p	3824.198199	2752.259102	3694.08542	4432.80306
ame-mir-125-5p	3689.419004	3988.989927	6659.229634	4993.56152
ame-mir-3785-3p	3185.734817	94025.05527	39880.14042	5409.07516
ame-mir-3747a-5p	3144.080951	16259.28439	23631.847	7549.47003
ame-mir-3791-5p	3032.011009	445.6036069	924.5486313	4596.22437
ame-mir-13a-5p	2794.889913	69221.21147	38041.87996	4571.32815
ame-mir-317-3p	2617.041659	9047.860278	13289.2923	6543.66166
ame-mir-71-3p	2429.97847	145820.5417	47684.43981	4108.25855
ame-mir-276-3p	2399.174258	302681.6849	158768.9272	3519.66825
ame-mir-3715-5p	2263.418513	12431.73583	11539.6901	6542.8371
ame-mir-13b-5p	2215.083843	63014.70576	21143.21109	3922.79023
ame-mir-252b-5p	2173.091733	51729.5319	108296.6503	360.716343
ame-mir-2-3-3p	2172.881772	29648.91883	26417.9722	5746.81254
ame-mir-276-5p	2122.726195	51057.51101	109297.5359	2516.06806
ame-mir-13b-3p	2099.605539	157249.2148	93245.28352	3096.14861
ame-mir-12-5p	2068.36442	102252.3051	60775.14859	5493.31877
ame-mir-275-3p	1940.665399	42308.42365	34438.06979	2517.19888
ame-mir-6040-3p	1637.69232	741.3819039	4353.57077	2615.19349
ame-mir-11-3p	1574.218134	15184.59974	24821.0787	1737.06072
ame-mir-993-3p	1565.503635	48421.51915	13183.78345	1947.8007
ame-bantam-3p	1458.501847	164172.8658	35963.04738	2115.40786
ame-mir-318-5p	1426.753137	12.30108802	0	2767.14779
ame-mir-190-5p	1260.603165	105569.6596	32546.44329	3989.52275
ame-mir-92a-5p	1210.842514	555.9353722	1238.653979	1044.87501
ame-mir-71-5p	1069.854002	20871.52667	17538.04437	1935.39348
ame-mir-31a-5p	870.8113972	225999.341	26968.76199	1959.14064
ame-mir-9a-3p	810.2788042	20202.93935	15050.96789	996.288865
ame-mir-3747a-3p	755.074559	2106.882621	4221.965424	1187.35796
ame-mir-279b-5p	705.2690731	665.1885993	1828.966728	2299.92172
ame-mir-9c-3p	692.114574	2147.531027	5920.465874	1845.10302
ame-mir-92c-5p	616.9511641	277.9145814	589.6326054	1274.23781
ame-mir-7-3p	562.428817	2168.116251	2583.930917	805.219768

ame-mir-8-5p	536.6344745	47926.90581	24462.27992	377.160764
ame-mir-8-3p	487.8495222	133958.1395	31440.61219	1424.82955
ame-mir-6045-3p	457.2025651	900.6919734	1667.898099	806.794513
ame-mir-279a-5p	431.2589777	4295.798261	5281.102178	593.678981
ame-mir-3747b-3p	377.7211152	31.61745001	261.5640812	364.436602
ame-mir-306-3p	372.2253441	676.6179567	1303.040673	308.526085
ame-mir-305-3p	331.7376751	673.3478906	1240.592405	290.243057
ame-mir-3756-5p	304.9059514	456.2708716	573.5866605	942.901911
ame-mir-283-5p	298.5639076	22651.82624	13044.49684	332.460229
ame-mir-281-5p	281.8159861	23716.28274	4524.785493	610.970602
ame-mir-6045-5p	276.4480626	52.10614722	108.637041	806.794513
ame-mir-252a-5p	273.683582	5115.186035	2517.184143	474.943185
ame-mir-305-5p	267.2331272	9777.656495	10938.25893	606.871847
ame-mir-6000a-5p	220.0300906	5.92460566	50.86248016	484.635903
ame-mir-6051-5p	207.3360469	1080.051714	1888.871895	546.809588
ame-mir-125-3p	201.5228868	156.6836717	416.9297322	554.840169
ame-mir-275-5p	199.0426051	320.7877735	757.6475045	213.724433
ame-mir-34-5p	190.7491632	1012.44105	1388.189671	320.58665
ame-mir-14-5p	182.4557213	187805.2746	3070.466202	3312.72871
ame-mir-33-5p	182.4557213	1528.459391	1044.25758	249.345172
ame-mir-9c-5p	173.0286435	601.4966553	900.0829403	589.407909
ame-mir-iab-4-5p	156.1118471	13789.51967	10860.39612	181.595924
ame-mir-965-3p	142.9903772	2220.671589	2423.509486	559.559499
ame-mir-3719-3p	141.2956764	544.5919466	498.4523056	228.676348
ame-mir-3763-5p	126.3762572	0	16.61507685	576.716725
ame-mir-29b-3p	124.4016282	9661.372943	3192.587017	201.850854
ame-mir-315-5p	121.3674421	33460.38455	5157.765626	333.039428
ame-mir-993-5p	121.1401623	4594.331984	1680.176311	106.728806
ame-mir-1175-3p	120.1948098	318.1792297	146.2849158	154.872778
ame-mir-14-3p	116.1081863	130294.9941	3404.429247	3965.77559
ame-mir-87-2-5p	116.1081863	5078.897825	1328.375394	225.598013
ame-mir-750-3p	115.8642615	13648.63604	2285.183916	488.912102
ame-mir-3723-3p	114.1299341	12.65093548	22.86478466	239.650231
ame-mir-6064-5p	107.3268949	151.9835605	357.7128311	251.44051
ame-mir-375-3p	99.52130253	9473.400017	4687.943934	166.230115
ame-mir-277-3p	98.73145093	2116.812231	1516.125763	212.028208
ame-mir-3786-3p	95.69356013	1129.121601	661.407867	194.087359
ame-mir-3759-3p	90.14610737	142.7862163	16.25387953	232.309167
ame-mir-7-5p	85.79422632	2844.661953	3572.241523	95.5345487
ame-mir-3756-3p	85.37366639	644.1157216	844.8033562	1004.01592
ame-mir-263b-5p	82.02305154	7145.985904	1128.36456	143.526787
ame-mir-3746-5p	81.67783668	115.4624853	341.7419216	287.844354
ame-mir-3759-5p	81.13149663	190.9075378	100.2322571	90.3424537
ame-mir-927a-5p	74.6409769	1328.148474	3362.060801	94.988637
ame-mir-996-5p	72.90937915	575.0285532	854.4896667	91.3352279
ame-mir-11-5p	69.11201565	927.3653582	1250.746063	109.940552
ame-mir-13a-3p	66.34753502	1441.367688	1722.152716	106.862217
ame-mir-283-3p	66.34753502	727.2157218	1046.749842	0
ame-mir-184-5p	63.10227516	1588.00361	1446.595278	154.872778
ame-mir-3743-3p	60.68372106	683.2804357	361.6818559	144.799752

ame-mir-6039-5p	56.54619462	842.0514108	1702.101339	0
ame-mir-307-3p	56.12103526	2067.60942	1099.968058	169.622566
ame-mir-2796-3p	51.83401174	2092.242089	251.1732321	74.2098726
ame-mir-33-3p	49.76065127	114.6707426	107.1672457	0
ame-mir-219-5p	48.78495222	49.09581308	21.99054289	0
ame-mir-750-5p	48.78495222	2240.796947	637.7257439	0
ame-mir-3049-5p	48.37841095	2383.530572	2357.264029	108.841147
ame-mir-2-1-5p	46.5923701	1005.468821	501.2525994	0
ame-mir-1000-5p	45.56836197	7768.867042	736.7234627	378.388801
ame-mir-3715-3p	43.19500978	244.9454153	269.9949989	98.9464969
ame-mir-3719-5p	30.7164514	116.1222709	88.61374322	0
ame-mir-3720-5p	29.61943528	266.4591396	206.5016695	0
ame-mir-193-3p	0	125.0266047	18.84903677	0
ame-mir-210-3p	0	17.93064478	43.98108579	0
ame-mir-2765-5p	0	457.4760815	229.6240958	0
ame-mir-3725-5p	0	11.11530747	11.22640328	0
ame-mir-3727-3p	0	852.2231059	717.4692277	0
ame-mir-3730-5p	0	20.84641832	18.55939436	0
ame-mir-3732-3p	0	15.16522195	24.17865661	0
ame-mir-3772-5p	0	9.072052418	0	0
ame-mir-3793-5p	0	584.1953754	203.0731615	0
ame-mir-3797-3p	0	3.155496493	0	0
ame-mir-3798-3p	0	10.92548248	0	0
ame-mir-6001-3p	0	19.21140512	32.98581434	0
ame-mir-6012-3p	0	40.13049069	14.66036193	0
ame-mir-6043-3p	0	123.5760654	26.94336787	0
ame-mir-6052-5p	0	19.63832523	17.59243432	0
ame-mir-6055-5p	0	40.94054424	15.97603544	0
ame-mir-6056-5p	0	7.443735317	19.17124252	0
ame-mir-6057-5p	0	5.806113547	0	0
ame-mir-6059-3p	0	8.797141738	0	0
ame-mir-927b-5p	0	11.26185817	15.03950922	0
ame-mir-929-3p	0	26.85327516	0	0
ame-mir-932-5p	0	75.47947611	147.0434301	71.2414777
ame-mir-981-3p	0	116.5855247	151.1264969	0
ame-mir-989-3p	0	391.7565861	160.7910663	0
ame-bantam-5p	0	92.59750054	28.64668423	0
ame-let-7-3p	0	52.98078612	117.1362918	0
ame-mir-1000-3p	0	9.570516836	0	0
ame-mir-100-3p	0	35.56244548	69.78332278	59.3678981
ame-mir-1175-5p	0	805.1775218	204.0764874	51.6242592
ame-mir-124-3p	0	1698.476723	61.49736238	92.5213997
ame-mir-124-5p	0	186.6250783	0	0
ame-mir-12-3p	0	43.72073454	27.02452259	0
ame-mir-133-3p	0	532.498897	47.60499548	0
ame-mir-133-5p	0	9.78558463	0	0
ame-mir-137-3p	0	205.3912667	152.0279532	0
ame-mir-137-5p	0	17.41834064	17.4458307	0
ame-mir-190-3p	0	15.96681226	0	0
ame-mir-2-1-3p	0	402.8399006	322.0337929	0

ame-mir-219-3p	0	7.115335229	0	0
ame-mir-2-2-3p	0	17.28009984	20.76884607	0
ame-mir-2-3-5p	0	1362.985155	655.4647819	71.2414777
ame-mir-252a-3p	0	36.28820967	0	0
ame-mir-252b-3p	0	47.77182033	463.7499299	0
ame-mir-263a-3p	0	270.3671006	95.85621261	0
ame-mir-263b-3p	0	43.06732576	19.17124252	0
ame-mir-278-3p	0	102.553636	62.3065382	0
ame-mir-278-5p	0	211.418265	73.14245789	0
ame-mir-2788-3p	0	171.6059261	44.25511124	0
ame-mir-2788-5p	0	114.6300455	0	0
ame-mir-2796-5p	0	25.51514742	0	0
ame-mir-281-3p	0	1378.951967	195.9933823	0
ame-mir-282-3p	0	18.93297896	0	0
ame-mir-282-5p	0	42.59920266	16.25387953	0
ame-mir-29b-5p	0	87.09170321	34.89166139	0
ame-mir-3049-3p	0	765.0764206	504.6829594	118.735796
ame-mir-307-5p	0	153.8838365	91.82016156	0
ame-mir-315-3p	0	111.5198639	0	0
ame-mir-316-3p	0	1584.449245	0	0
ame-mir-316-5p	0	10181.08534	25.20264466	560.326229
ame-mir-318-3p	0	42.43875368	255.5624109	0
ame-mir-31a-3p	0	5.080349354	0	0
ame-mir-34-3p	0	94.34934514	117.1362918	0
ame-mir-3477-3p	0	5.131666014	12.58717943	0
ame-mir-3477-5p	0	285.9071065	181.2553839	0
ame-mir-3720-3p	0	56.50592649	48.06504375	0
ame-mir-3770-3p	0	5.462741241	18.75895774	0
ame-mir-3770-5p	0	10.92548248	18.75895774	0
ame-mir-3773-3p	0	16.0343252	0	0
ame-mir-3773-5p	0	49.79079932	28.97978521	0
ame-mir-3783-3p	0	95.94848659	0	0
ame-mir-3783-5p	0	4.305380808	0	0
ame-mir-3785-5p	0	66.24038273	37.58172145	0
ame-mir-3786-5p	0	128.4044342	136.595103	0
ame-mir-6005-3p	0	171.422831	119.2615946	0
ame-mir-6005-5p	0	101.0726821	77.97873492	0
ame-mir-6037-3p	0	305.3214883	355.2188844	0
ame-mir-6037-5p	0	80.08432479	88.80472111	0
ame-mir-6039-3p	0	165.7711396	467.2990365	0
ame-mir-6042-3p	0	8.268199672	0	0
ame-mir-6042-5p	0	5.512133114	0	0
ame-mir-6044-3p	0	352.6469606	313.1302945	106.557766
ame-mir-6044-5p	0	160.0403093	236.4453244	0
ame-mir-6048-3p	0	9.167547706	0	0
ame-mir-6048-5p	0	3.819811544	0	0
ame-mir-6053-3p	0	23.9172291	0	0
ame-mir-6053-5p	0	216.0797939	138.7736533	0
ame-mir-6060-3p	0	24.53287414	70.20455008	0
ame-mir-6060-5p	0	145.1528387	186.0420577	0

ame-mir-6061-3p	0	61.55215311	25.23809142	0
ame-mir-6061-5p	0	13.78033279	0	0
ame-mir-6065-3p	0	178.5761897	163.9645742	0
ame-mir-6065-5p	0	15.27924618	0	0
ame-mir-6066-3p	0	6.912039937	0	0
ame-mir-6066-5p	0	6.912039937	0	0
ame-mir-6067-3p	0	9.304669146	0	0
ame-mir-6067-5p	0	125.6130335	89.46579844	76.1126899
ame-mir-87-1-3p	0	34.11091709	34.89166139	0
ame-mir-87-1-5p	0	773.6646302	490.975521	0
ame-mir-87-2-3p	0	5.806113547	0	0
ame-mir-927a-3p	0	355.6244548	578.2046745	0
ame-mir-965-5p	0	216.8950463	286.4668423	0
ame-mir-971-3p	0	6.286146557	15.69928522	0
ame-mir-971-5p	0	6.857614426	11.77446391	0
ame-mir-980-3p	0	237.1392306	60.85754894	0
ame-mir-980-5p	0	21.09779632	14.4898926	0
ame-mir-iab-4-3p	0	109.2915491	82.0980268	0

Table S2 - Sequence and read numbers of the novel miRNAs described from *A. mellifera* embryogenesis libraries.

miRNA	Reads Number	Sequence (5' - 3')
ame-miR-92c-3p	736069	AAUUGCACUCGUCCCGGCCUGC
ame-miR-14-5p	260302	GGGGGUGAGAAACUGGCUUGGCU
ame-miR-71-3p	220692	UCUCACUAUCUUGUCUUUCAUC
ame-miR-13a-5p	111363	ACAUCAAAUUGGUUGUGGAAUG
ame-miR-276-5p	96325	AGCGAGGUAUAGAGUUCCUACG

ame-miR-13b-5p	75766	UCGUCAAAUUGGUUGUGGCGUG
ame-miR-8-5p	64556	CAUCUUACCGGGCAGCAUUAGA
ame-miR-6040-5p	46863	CCUCAGUCCAGCCUUGUACUGUG
ame-miR-281-5p	35616	AAGAGAGCUAUCCAUCGACAGU
ame-miR-9a-3p	29630	AUAAAGCUAGGUUACCGGAGUUA
ame-miR-279c-5p	26796	GACGGGUGCACUCUGGUAUCAUG
ame-miR-2-2-5p	18955	CCCAUCAGAGUGGUUGUGAUGUG
ame-miR-279a-5p	8140	AAUGAGUGAAGGUCUAGUGCAC
ame-miR-87-2-5p	7564	GGGCCUGACUCUUUGCUCUGCC
ame-miR-993-5p	6255	UACCCUGUAGAUCCGGGCUUUUG
ame-miR-317-5p	5498	AGGGAGCCACUCUGCGUUCACU
ame-miR-750-5p	4555	AGUUGGAAGUGAGGAUCUAGGCA
ame-miR-7-3p	3619	CAAGGAAUCACUAAUCAUCCU
ame-miR-2944-5p	3299	CGGAGGAACUCUUGCUGUGAUAU
ame-miR-3747a-3p	3208	UCGUUGGAAAAAAGUCUUGGAGA
ame-miR-184-5p	2566	CCUUAUCAUUCUCCUGUCCGGU
ame-miR-279b-5p	2450	ACGAAGUAUUCAUCUAGUGCAACG
ame-miR-2-3-5p	2147	CCAUCAAAGUUGGUUUGUCAUA
ame-miR-316-3p	1943	CCAGCAAAGGGGAACAGGCCGA
ame-miR-11-5p	1941	UGGAGCUCUGGCUGUGACUUGUG
ame-miR-306-3p	1938	CAAAGCAGCUUGGUACCUAACU
ame-miR-3786-3p	1923	UCUGUAUGGCUCAGGACGAUAC
ame-miR-3756-3p	1797	CUUCUUAUGAUAGAAAUCAGCU
ame-miR-6051-5p	1761	AAUCAGAGCUUGUGUAAUUUUC
ame-miR-9c-5p	1752	UCUUUGGUGAGCUAACUGUAUGG
ame-miR-1175-5p	1650	AAGUGGAGAAGUGGUCUUUACGCU
ame-miR-3791-5p	1616	AUGAAAUCUCGUCCGGUGUGGU
ame-miR-6045-3p	1586	CAUGUCGCCGUCCAGUCAGAGUCU
ame-miR-92a-5p	1497	AGGCCGAGAUUUGUGACAAUGUU
ame-miR-283-3p	1430	CAGGAUUCUUGCUGGUAUCCGG
ame-miR-2-1-5p	1417	CUCACAAAGUGGUUGUGAUAUG
ame-miR-305-3p	1341	CGGCACCUGUUGGAGCGCAAUUC
ame-miR-87-1-5p	1263	ACGCCUGAAAUUUGCUUUUUACCU
ame-miR-996-5p	1048	GGCGAGUAUGAAUGUGGUGCACG
ame-miR-275-5p	788	CGCGUUACUCGGGUACUUUAGGCU
ame-miR-927a-3p	722	CAAAGCGUUUGAAAUCUGAAAC
ame-miR-318-5p	498	GGAUGAACGUGAUCCUGUUUGACG
ame-miR-125-3p	486	UCACAGGCUAGAUUCUCUGGUA
ame-miR-6044-3p	484	UGCUAAUCUAAGAGUCUCGGGUU
ame-miR-3715-3p	441	UAUUAUGCUCCGUUUAUCGUUG
ame-miR-3746-5p	436	UUCUUUGUUUUUUUUUCUUUUU
ame-miR-263a-3p	374	CGUGGAGCUCCCGUGUCAUACA
ame-miR-6039-3p	366	GUAGACACACGUGUUGGACCU
ame-miR-307-5p	331	ACUCACUCAACCUGGGUGUGAU
ame-miR-3759-5p	295	GGCUAGUUAUGACGGAGGGUC
ame-miR-278-5p	295	CCGGAUGAGGUCUUCAUCGACC
ame-miR-3719-5p	269	ACCGAAAUGUCGCGCAAACUGU
ame-miR-3747b-3p	227	CUGCUGGAAAAAAAUCUCGCGGA
ame-miR-33-3p	207	CAAUACUUCUACAGUGCAACUC
ame-miR-252b-3p	199	AUCUUGGCACGAUUACUUUUCA

ame-miR-124-5p	198	UGCGUUCACUGCGGGCUUCCAUG
ame-miR-34-3p	177	CGACCGCUAUCGGCACUGCAAUU
ame-miR-2788-5p	169	UGGGGUUUCUAUGCGGCAUGUGCC
ame-miR-iab-4-3p	156	UCCGGUAUACCUUCAGUAUACG
ame-miR-3720-3p	136	AGAGUCAUAGUUACGCUGAGUAUG
ame-miR-29b-5p	134	ACUGACUUCUAUGCGUGCUGGG
ame-miR-3785-5p	134	UCUCACGACGUGAUAGCGGCGCC
ame-miR-6037-5p	127	GUAAUAGUCCAUAGAGCUUCA
ame-miR-315-3p	126	CUUUCGGGCAAUAAUCAUAAUC
ame-bantam-5p	121	CUGGUUUUCACAAUGAUUUGACAG
ame-let-7-3p	120	AAGUCCACUGUACAACUUGCU
ame-miR-100-3p	82	CAAGCUCCUAUCUACCGGUACA
ame-miR-3773-5p	69	UGGCAAGUGCUGUUGCAAACUGUC
ame-miR-3723-3p	66	CACGACGAUAUCUCCGGAACCGGA
ame-miR-263b-3p	61	CGUGGAUCUUGCAAUGCCAUC
ame-miR-12-3p	59	CAGUACUUGUGUUAUACUUACG
ame-miR-252a-3p	50	CUGCUGCUCAAGUGCUUAUCA
ame-miR-2796-5p	45	AGGGGUUUCUAUCGGCCUCCAG
ame-miR-6060-3p	44	GUUGCCCCCUUGUCCCCGAGA
ame-miR-929-3p	37	CUUCCUAAUAGAGUCAGGCUG
ame-miR-137-5p	31	ACGCGUAUUCUUGGGGAAUUA
ame-miR-980-5p	30	CGGCUCGUCAUCGGGCACUUUGC
ame-miR-6053-3p	29	GCUCGCGCAGUGUUUCGUAAAG
ame-miR-282-3p	24	ACAUAGCCUAGAAUAGGUUAGGU
ame-miR-190-3p	22	ACCAGGAAUCAAACAUAUUAUU
ame-miR-3725-5p	22	AAGGAAGAGAGAGAGAGAG
ame-miR-971-5p	18	CACUUUAAGUUCGAAUACCAAG
ame-miR-6065-5p	16	CGGGGGGAACCGCAUUCCAGAGU
ame-miR-6061-5p	15	ACGUGGUACAUGCCGAACGUUCGU
ame-miR-3770-3p	14	AAUGCAUUGUAAGCAGGAUAGU
ame-miR-3772-5p	12	GGGUUCGAUUCCCAGGGCU
ame-miR-1000-3p	12	CUGGUGUGAUGGGACAUUGUCC
ame-miR-133-5p	12	AGCUGGUUGAACACGGGUCAAAU
ame-miR-3477-3p	12	CAUAUUACCUCGUGGGGUUUCC
ame-miR-6067-3p	10	CAAAAAGCGGGAUUCGCUUUU
ame-miR-219-3p	10	AGAAUUGUGUGGGGACAUCAGC
ame-miR-6066-3p	8	CCUCUUCUCUCUCGUCCUC
ame-miR-31a-3p	7	GCUGUGUCACAUCCAGCCAACC
ame-miR-3783-5p	7	CCACCAAACAAGUGAGAGUGUG
ame-miR-3797-3p	6	AGAAAGAAAGGAAGAAAUA
ame-miR-6042-5p	6	ACCACUGUACUAUCGUCGCCUGC
ame-miR-6048-5p	5	AAUCAGCGGUCGAACCUCGUCGAU

Table S3(a) - miRNA-target interactions predicted by RNAhybrid in both *A. mellifera* and *D. melanogaster*. miRNAs were classified according to the miRNA family (first row) and only miRNAs from families conserved in both species were considered for further analysis. Third row: number of genes predicted for ech miRNA. Fourth row: Number of predicted targets with ortholog in the other specie. miR-34 presented one of the highest number of predicted targets in both species.

miRNA_family	miRNA	N_Targets_Apis	N_Targets_Drosophila	*Apis* ∩ *Drosophila*
mir-34	miR-34-5p	9440	12624	2881

mir-210	miR-210-3p	9618	11987	2773
mir-31	miR-31a-5p	8825	11393	2388
mir-317	miR-317-3p	8891	11256	2335
mir-124	miR-124-3p	8641	11531	2305
mir-282	miR-282-5p	8998	10384	2129
mir-275	miR-275-3p	8206	10395	1972
mir-980	miR-980-3p	7178	11568	1888
mir-278	miR-278-3p	7942	10857	1820
mir-316	miR-316-5p	7356	7803	1244
mir-932	miR-932-5p	6710	7680	1212
mir-10	miR-993-3p	6632	8486	1202
mir-25	miR-92a-3p	5574	9882	1185
mir-305	miR-305-5p	6035	8334	1144
mir-184	miR-184-3p	6820	7877	1110
mir-263	miR-263a-5p	4773	8529	933
let-7	let-7-5p	5663	6623	923
mir-9	miR-9a-5p	5479	7285	912
mir-33	miR-33-5p	4127	8173	873
mir-375	miR-375-3p	7030	5899	868
mir-133	miR-133-3p	4842	8548	801
mir-965	miR-965-5p	6706	5137	735
mir-190	miR-190-5p	8032	3750	724
mir-9	miR-9b-5p	3802	7615	661
mir-263	miR-263b-5p	3419	8033	602
mir-981	miR-981-3p	4534	6371	599
mir-971	miR-971-3p	5414	4663	591
mir-12	miR-12-5p	4732	5180	580
mir-277	miR-277-3p	4443	5375	565
mir-7	miR-7-5p	5238	4759	553
mir-965	miR-965-3p	4011	6649	534
mir-14	miR-14-3p	4487	4711	467
bantam	bantam-3p	5221	3811	450
mir-1000	miR-1000-5p	3193	6008	445
mir-10	miR-125-5p	3617	6286	430
mir-929	miR-929-5p	3890	4752	385
mir-11	miR-11-3p	4073	3647	344
mir-10	miR-10-5p	2782	5486	317
mir-10	miR-100-5p	3930	3945	315
mir-iab-4	miR-iab-4-5p	3249	3151	248
mir-315	miR-315-5p	2589	3534	198
mir-137	miR-137-3p	2706	2715	176
mir-2	miR-13a-3p	2201	3662	176
mir-8	miR-8-3p	2340	2846	156
mir-219	miR-219-5p	2899	1990	128
mir-9	miR-9c-3p	3318	1299	89
mir-216	miR-283-5p	784	3063	50
mir-9	miR-79-3p	708	2560	44

Table S3(b)- Description of the distribution of targets predicted for each miRNA in *A. mellifera* , *D. melanogaster* and for the intersection between *A. mellifera* and *D. melanogaster* .

N_Targets/miRN	*Apis*	*Drosophila*	*Apis* ∩ *Drosophila*
**Total**	251098	318112	43460
**Average**	5231	6627	905
**St.Dev**	1889	2545	586
**Minimum**	708	1299	44
**First Quartile**	3568	3912	375
**Second Quartile**	4808	6329	632
**Third Quartile**	6873	8497	1189
**Maximum**	9618	12624	2881

TableS4 - Gene Ontology analysis of the 2881 genes predicted as *miR-34-5p* in both *A. mellifera* and *D. melanogaster* . This chart was generated by ClueGO plugin in the Cytoscape platform

GOID	GOTerm	GOLevels	Nr. Genes	% Associated Genes	Term PValue	Term PValue Corrected with Bonferroni step down
GO:0009653	anatomical structure morphogenesis	[2, 3]	748	37.47495	1.21E-87	1.57E-84
GO:0007389	pattern specification process	[3, 4]	274	44.99179	4.85E-46	6.01E-43
GO:0050794	regulation of cellular process	[2, 3]	1117	35.449062	9.37E-126	1.22E-122
GO:0048731	system development	[3, 4]	916	36.262867	8.65E-103	1.12E-99
GO:0007275	multicellular organismal development	[3]	1058	33.61932	2.35E-98	3.05E-95
GO:0048869	cellular developmental process	[3]	899	35.589867	8.11E-95	1.05E-91
GO:0030154	cell differentiation	[4]	859	35.39349	6.58E-88	8.53E-85

GO:0007154	cell communication	[3]	705	36.969063	1.77E-78	2.29E-75
GO:0048468	cell development	[3, 4, 5]	635	38.34541	6.57E-77	8.49E-74
GO:0007399	nervous system development	[4, 5]	644	38.061466	1.36E-76	1.76E-73
GO:0048513	organ development	[3, 4, 5]	597	39.019608	4.36E-75	5.63E-72
GO:0019222	regulation of metabolic process	[2, 3]	678	36.49085	4.28E-72	5.52E-69
GO:0009888	tissue development	[3]	514	40.250587	8.53E-69	1.10E-65
GO:0060429	epithelium development	[4]	476	41.319443	1.58E-67	2.04E-64
GO:0048518	positive regulation of biological process	[1, 2, 3]	497	40.340908	1.17E-66	1.51E-63
GO:0022008	neurogenesis	[5, 6]	567	38.156124	1.35E-66	1.74E-63
GO:0031323	regulation of cellular metabolic process	[3, 4]	604	37.14637	2.37E-66	3.05E-63
GO:0048699	generation of neurons	[6, 7]	417	43.4375	5.69E-66	7.31E-63
GO:0060255	regulation of macromolecule metabolic process	[3, 4]	609	36.708862	1.05E-64	1.34E-61

GO:0080090	regulation of primary metabolic process	[3, 4]	585	37.048763	1.80E-63	2.31E-60
GO:0009887	organ morphogenesis	[3, 4, 5, 6]	420	42.12638	8.38E-62	1.07E-58
GO:0035295	tube development	[3, 4]	387	43.58108	2.24E-61	2.87E-58
GO:0048522	positive regulation of cellular process	[2, 3, 4]	441	41.09972	2.64E-61	3.38E-58
GO:0048729	tissue morphogenesis	[3, 4]	350	45.572918	4.53E-61	5.79E-58
GO:0030182	neuron differentiation	[5, 7, 8]	383	43.62187	7.82E-61	9.99E-58
GO:0009791	post-embryonic development	[3, 4]	339	46.122448	1.24E-60	1.59E-57
GO:0002009	morphogenesis of an epithelium	[4, 5]	342	45.47872	2.48E-59	3.16E-56
GO:0035239	tube morphogenesis	[3, 4, 5]	306	47.589424	2.81E-58	3.58E-55
GO:0002165	instar larval or pupal development	[4, 5]	312	46.77661	2.53E-57	3.22E-54

GO:0032989	cellular component morphogenesis	[3, 4]	395	41.887592	4.52E-57	5.75E-54
GO:0007444	imaginal disc development	[3, 4, 5, 6]	332	45.170067	1.18E-56	1.51E-53
GO:0048569	post-embryonic organ development	[3, 4, 5, 6]	286	48.067226	1.70E-55	2.16E-52
GO:0007165	signal transduction	[3, 4]	527	36.827393	2.08E-55	2.64E-52
GO:0009886	post-embryonic morphogenesis	[3, 4, 5]	274	48.495575	4.31E-54	5.46E-51
GO:0010468	regulation of gene expression	[4, 5]	492	37.5	4.32E-54	5.47E-51
GO:0060562	epithelial tube morphogenesis	[4, 5, 6]	285	47.5	6.17E-54	7.81E-51
GO:0048707	instar larval or pupal morphogenesis	[4, 5, 6]	265	48.62385	1.47E-52	1.86E-49
GO:0050793	regulation of developmental process	[2, 3]	317	44.335663	9.16E-52	1.16E-48
GO:0007560	imaginal disc morphogenesis	[4, 5, 6, 7, 8]	258	48.863636	1.16E-51	1.47E-48

GO:0051171	regulation of nitrogen compound metabolic process	[3, 4]	476	37.362637	1.31E-51	1.65E-48
GO:0048666	neuron development	[4, 5, 6, 8, 9]	329	43.46103	2.17E-51	2.73E-48
GO:0031326	regulation of cellular biosynthetic process	[4, 5]	452	38.04714	2.42E-51	3.05E-48
GO:0051252	regulation of RNA metabolic process	[4, 5, 6, 7]	429	38.82353	2.73E-51	3.44E-48
GO:0007552	metamorphosis	[3, 4]	272	47.38676	3.75E-51	4.72E-48
GO:0019219	regulation of nucleobase-containing compound metabolic process	[4, 5]	442	38.2684	6.34E-51	7.97E-48
GO:0016070	RNA metabolic process	[4, 5, 6]	571	34.753498	9.86E-51	1.24E-47
GO:1901362	organic cyclic compound biosynthetic process	[4]	501	36.35704	2.09E-50	2.63E-47

GO:2000112	regulation of cellular macromolecule biosynthetic process	[5, 6]	434	38.339222	3.50E-50	4.39E-47
GO:0000902	cell morphogenesis	[4, 5]	343	42.085888	1.00E-49	1.26E-46

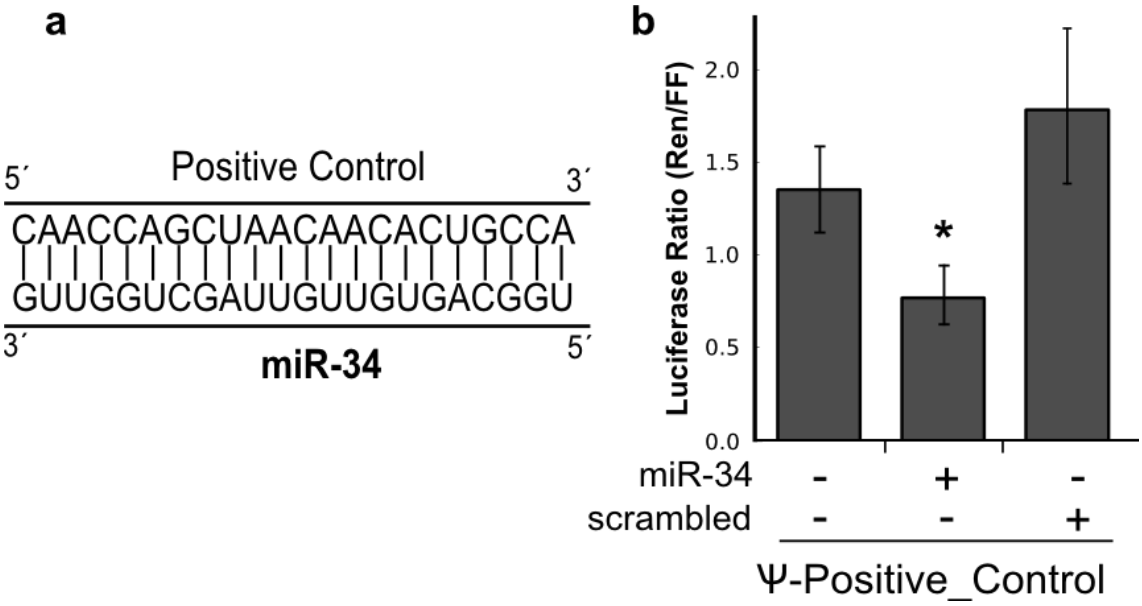

Figure S2 – The positive-control vector showed significantly reduced luciferase activity in the presence of the *miR-34-5p* mimic. Dual-luciferase reporter analysis of *miR-34-5p* target interactions. Changes in expression ratio of Firefly (Fluc) and chimeric *Renilla* luciferase (Rluc) containing perfectly matched target sites for *miR-34-5p* are treated with and without *miR-34-5p* mimic and scrambled RNA are shown. Two sample *t*-tests were used to test the effect of mimic *miR-34-5p* in the honey bee 3'UTRs, *: $p<0.05$. Schematic representation of positive control, perfect match 22pb *miR-34-5p* sequence (a). The positive control (b: Positive Control; $p=0.0005$) is significantly down-regulated by *miR-34-5p*.

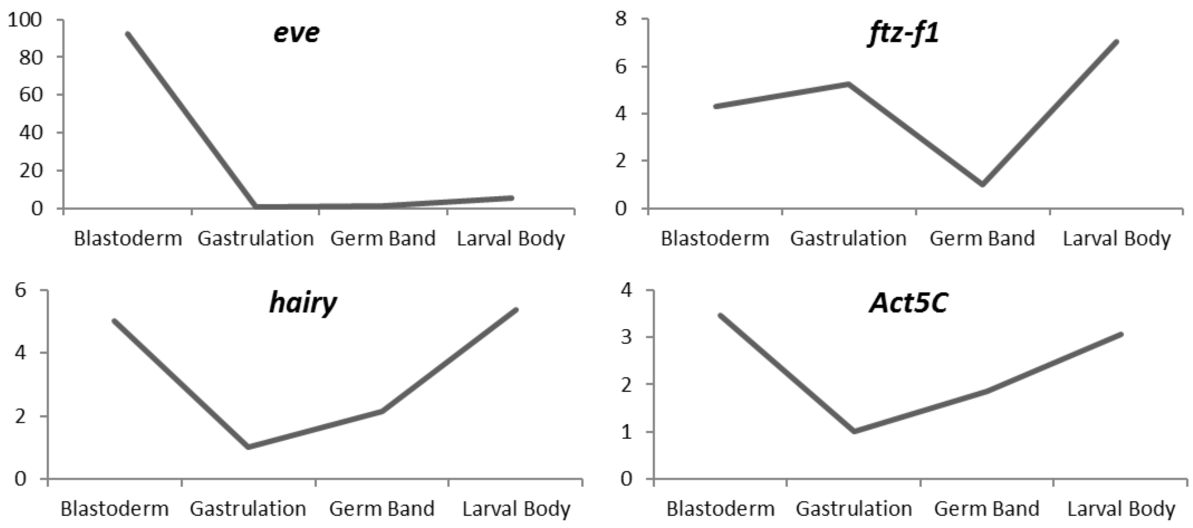

Figure S3 – Expression profiles of *ame-miR-34-5p* targets in honey bee female embryos. Transcript levels of *miR-34-5p* targets *eve*, *h*, *ftz-f1* and *act5C* by qPCR. The y-axis refers to transcript levels and x-axis, to the embryonic stages.

Table S5 - Novel miRNAs and forward primers sequences used in RT-qPCR reactions.

Novel miRNA	miRNA Sequence (5'- 3')	Forward Primer (5'- 3')	Temp (oC)
**ame-mir-92c-3p**	AAUUGCACUCGUCCCGGCCUGC	AATTGCACTCGTCCCGGCCTGC	60
**ame-mir-184-5p**	CCUUAUCAUUCUCCUGUCCGGU	CCTTATCATTCTCCTGTCCGGT	60
**ame-mir-306-3p**	CAAAGCAGCUUGGUACCUAACU	CAAAGCAGCTTGGTACCTAACT	60
**ame-mir-34-3p**	CGACCGCUAUCGGCACUGCAAUU	CGACCGCTATCGGCACTGCAATT	60

Supplementary File S1

Luciferase reporter assay method

We used the psiCHECK2 vector to perform the luciferase-reporter assays. psiCHECK2 vector contains the coding region for two luciferases: from Firefly (Insecta) and from Renilla (Cnidaria). The Firefly luciferase is used as an internal control. The Renilla luciferase contains the multiclonal sites at the 3' end, after the stop codon. The putative 3' UTR was then inserted in this multiclonal site what means that the Renilla luciferase transcripts were under control of our putative target region.

Gene structures and the predicted binding sites were annotated and visualized using the program Unigene (Okonechnikov *et al.* 2012). We designed primers flanking the binding-site regions in the 3' UTR of each candidate gene. The restriction sites recognized by Xho-I and Not-I were added to the 5' end of the forward and reverse primers, respectively. Further, the extension sequence 'CAGTGACT' was added to the 5' end of both forward and reverse primers to provide space for the enzyme anchoring (**Table S2**).

The designed primers were used to amplify miRNAs binding sites-containing regions of each candidate gene in a PCR reaction, using embryo cDNAs as a template. In addition, we designed control sequences (forward and reverse primers) containing one perfect match for the entire mir-34 sequence. These long primers (approximately 100 bp) were annealed in a buffer (100 mM Tris Hcl pH 7.5, 1 M NaCl, 10 M EDTA) for 40 minutes at 90 ºC, followed by 2 hours at room temperature (RT). After the PCR amplification (3' UTRs) or the annealing procedure (control sequence), the samples were loaded into a 1% agarose gel and the fragments were purified using the QIAquick Gel Extraction Kit. Both PsiCHECK2 vector (2 ug) and purified fragment (700 ng) were digested by Not-I (1.5 µL; 20.000U/mL; New England Biolabs) and Xho-I (2.5 µL; 5.000 U/mL; New England Biolabs) enzymes in a reaction with 1.5 µL BSA, 4.0 µL Buffer 4 (New England Biolabs) and water up to 40 µL. The digestion reactions were incubated for 4 hours at 37 ºC, followed by 30 minutes at 65 ºC for enzymes inactivation. The digested

PsiCHECK2 vector was dephosphorylated by rAPid Alkaline Phosphatase (Roche) adding 3 μL rApid enzyme and 6 μL rApid enzyme buffer. The dephosphorylation reaction was incubated for 2 hours at 37 ºC, then 15 minutes at 75 ºC for enzyme inactivation. A ligation reaction was performed using 1.5 μL T4 ligase (New England Biolabs), 1.5 μL T4 Buffer, 150 ng of digested UTRs fragments and 50 ng of digested and dephosphorylated psiCHECK-2. After adding the ligation mix, the samples were incubated for 16 hours at 4 ºC.

Chemically competent cells (One Shot® TOP10 Chemically Competent *E. coli,* Invitrogen) were mixed with 10 μL ligation reaction and placed on ice for 30 minutes. The bacteria were then transfected with PsiCHECK2+3' UTR vectors through heat-shock (42 ºC for 30 s, immediately followed by 2 minutes on ice). Then, 250 μL SOC medium (Invitrogen) was added to the transformed bacteria, which were incubated for 1 hour at 37 ºC and 225 rpm. Transformed bacteria were plated in solid LB + Ampicilin (50 ng/μL) and incubated for 16 hours at 37 ºC.

The colonies were screened for psiCHECK2+3'UTR using PCR reactions. Ten μl of PCR reaction mix (0.4 μL 50mM MgCl2, 0.2 μL 100 μM primer forward, 0.2 μL 100 μM primer reverse, 0.1 μL Taq DNA Polymerase and water up to 10 μL) were loaded in each well of a 96-wells plate. A pipette tip was rubbed on a new LB plate (called a 'master plate') and then placed into the PCR reaction mix, following which it was used to touch each colony. The master plate was used as a backup for the colonies tested in the PCR amplification and was placed in the incubator (37 ºC) for 16 hours. The PCR amplification was performed for 40 cycles (92 ºC for 30 s, 60 ºC for 30 s, 72 ºC for 1 minutes) using primers flanking PsiCHECK2 multi-cloning region. The samples were loaded to a 1% agarose gel (2.5 mg agarose, 250 mL TAE1x Buffer) and visualized in the transilluminator (Safe Imager™ 2.0 Blue Light, Invitrogen). We cultivated the colonies positive for the PsiCHECK2+3'UTR vector by touching them with a pipette tip (in the master plate) and placing the pipette tip in a falcon with 5 mL of liquid LB + Ampicilin (50 ng/μL). The falcons were incubated for 16 hours at 28ºC and 200 rpm. The PsiCHECK2+3'UTR

vectors were then purified using the QIAgen Miniprep Kit and sequenced to confirm the identity of the fragment.

For the luciferase-reporter assays, we used COS-7 cells, which are derived from the kidney of African Green Monkey *Cercopithecus aethiops.* COS-7 cells were maintained in DMEM media (Dulbecco's Modified Eagle Medium, Invitrogen), containing 10% FBS (fetal bovine serum, Sigma-Aldrich) heat-inactivated (30 minutes, at 56 ºC) and Penicillin/Streptomycin (5000 Units/mL and 5000 µg/mL respectively), at 37 ºC and 5% CO_2.

Three hours prior to transfection, COS-7 cells were plated to 24-well plates ($0.5x10^5$ cells/well).COS-7 cells were then co-transfected for 24 hours with PsiCHECK2 (containing or not the candidates 3' UTR) (Promega, 200 ng/well), miRIDIAN mimic mir-34 or scrambled sequence (Dharmacon, 40 pmol/well) using Lipofectamine® LTX with Plus™ Reagent (Invitrogen, 3 µl/well) according to the manufacturer's instructions. For each gene, three experimental conditions were tested: only the vector, the vector and the mimic miRNA, and the vector and the scrambled sequence (**Figure 1**). Twenty-four hours post-transfection, COS-7 cells were washed with phosphate-buffered saline (PBS) and lysed in 300 µL Passive Lysis Buffer (Promega) at room temperature for 10 minutes at 200 rpm. Twenty µL of cell lysates was transferred to a 96-well plate (Lumitrac 2000, Greiner Bio One). Dual-Luciferase Reporter Assay System (Promega) was used to determine Firefly and Renilla luciferase activities. Using the micro injectors of POLARstart OPTIMA luminescence counter (BMG Labtech), 100 µL of luciferase assay reagent II was added to each well and the Firefly luciferase activity was measured. In sequence, 100 µl Stop&Glo reagent were loaded in each well Renilla luciferase activities were measured. The ratio between Renilla and Firefly luciferase activities was calculated for each experiment and the results were reported as the average of six replicates assays.

In our experiments, firstly the Firefly luciferase activity was measured (the Firefly luciferase is not under control of our putative target). We did not expected changes under the tested

conditions. Secondly, we measured the Renilla luciferase activity level. The Renilla luciferase activity was divided by the Firefly luciferase activity, in order to normalize the data based on the transfection efficiency.

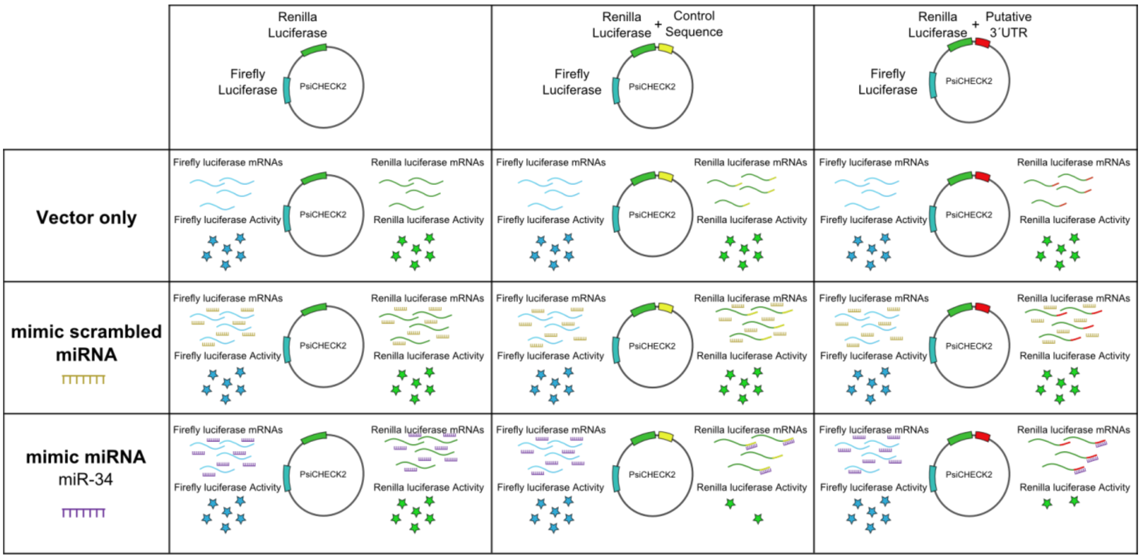

Figure 1 – Experimental conditions for luciferase-reporter assay. The Control Sequence contains two perfect matches for the entire miRNA. In the cells transfected with PsiCHECK2+3'UTR or Control Sequence, a decrease in Renilla activity in the presence of the mimic miRNA is expected.

References

Okonechnikov K., Golosova O., Fursov M. & team U. (2012) Unipro UGENE: a unified bioinformatics toolkit. Bioinformatics 28, 1166-7.

Table S6a - Primers used in luciferase assay in the amplification of 3'UTR (MREs). Genes name, primers name and sequence, fragment size, annealing temperature, access number of *A. mellifera* . The sequence 'CAGTGACT' corresponds to an extension for proper restriction enzyme anchoring and the sequences 'CTCGAG' and 'GCGGCCGC' correspond to the recognition sequence of the restriction Xho-I and Not-I, respectively.

Gene	Region	Primers name	Primers Sequence (5'- 3')	Fragment Size (pb)	Temp (oC)	*A. mellifera* Access Number
		ameve_34-5p_XhoI_F1	CAGTGACT/CTCGAG/TGAAGAAGCCGAGAACCTTT	121	59	GB49029
eve	3'UTR	ameve_34-5p_NotI_R2	CAGTGACT/GCGGCCGC/ACATTCGCATTCAGTCTCGAT			
		amftz-f1_34-5p_XhoI_F1	CAGTGACT/CTCGAG/TTGTCAATGTGGACCTCAAGT	319	58	GB42142
Ftz-f1	3'UTR	amftz-f1_34-5p_NotI_R4	CAGTGACT/GCGGCCGC/TTTGTTAACCACCCTTTGTACTGT			
		hairy_34-5p_XhoI_F1	CAGTGACT/CTCGAG/GGGTGGGTGAAAGGAAGTG	169	60	GB47799
hairy	3'UTR	hairy_34-5p_NotI_R2	CAGTGACT/GCGGCCGC/ATACGGTTCCCCCTTAACGA			
		act5C_utr_R4	CAGTGACT/CTCGAG/GACGATTCTTTCTCCGTCGT	537	58	GB44311
act5C	3'UTR	act5C_utr_F1	CAGTGACT/GCGGCCGC/TGTCTTTAAATATCGGCGGAGT			
		amwg_34-5p_XhoI_F1	CAGTGACT/CTCGAG/GTTGCGTTCGGTTTCGAC	92	60	
wg	3'UTR	amwg_34-5p_NotI_R2	CAGTGACT/GCGGCCGC/GCGTAATACTGAGAGACAATACAGGA			GB45510

TableS6b - Sequence of the mimic miRNAs used in luciferase assay

	mimic sequence
**scrambled**	ATTCACTTAGCAGTCAACCTAC
**miR-34**	TGGCAGTGTTGTTAGCTGGTTG

TableS7 - Primers used to amplify coding regions of gene eve, ftz-f1, h, act5C by RT-qPCR. Genes name, primers name and sequence, fragment size, annealing temperature, access number of *A. mellifera* .

Gene	Region	Primers name	Primers Sequence (5'- 3')	Fragment Size (pb)	Temp (oC)	*A. mellifera* Access Number
eve	CDS	ameve_CDS_F1<br>ameve_CDS_R2	AATTACGTGAGCAGGCCAAG<br>GCCATCCTTTGACGCTTATC	116	60	GB49029
Ftz-f1	CDS	FTZ-F<br>FTZ-R	GACTGGGCAAGGAATTCTGT<br>CCATTATGAAGCGTAGTCTCA	152	60	GB42142
hairy	CDS	amhairy_CDS_F1<br>amhairy_CDS_R2	TATGGAAAAGCGGAGACGAG<br>GTTTCACCGTCATCTCCAGAA	137	60	GB47799
act5c	CDS	amAct5C_CDS_F1<br>amAct5C_CDS_R2	AGGCCAATCGTGAAAAGATG<br>CCGGTAGTACGTCCAGAAGC	112	60	GB44311
rp49	CDS	amrp49-F<br>amrp49-R	CGTCATATGTTGCCAACTGGT<br>TTGAGCACGTTCAACAATGG	150	60	GB47227
